# New mutations and an updated database for the patched‐1 (*PTCH1*) gene

**DOI:** 10.1002/mgg3.380

**Published:** 2018-03-25

**Authors:** Marie G. Reinders, Antonius F. van Hout, Betûl Cosgun, Aimée D. Paulussen, Edward M. Leter, Peter M. Steijlen, Klara Mosterd, Michel van Geel, Johan J. Gille

**Affiliations:** ^1^ Department of Dermatology Maastricht University Medical Center Maastricht The Netherlands; ^2^ GROW Research School for Oncology and Developmental Biology Maastricht University Medical Center Maastricht The Netherlands; ^3^ Clinical Genetics Maastricht University Medical Center Maastricht The Netherlands; ^4^ Department of Clinical Genetics VU University Medical Center Amsterdam The Netherlands

**Keywords:** Basal cell nevus syndrome, BCNS, Gorlin syndrome, mutation database, *PTCH1*

## Abstract

**Background:**

Basal cell nevus syndrome (BCNS) is an autosomal dominant disorder characterized by multiple basal cell carcinomas (BCCs), maxillary keratocysts, and cerebral calcifications. BCNS most commonly is caused by a germline mutation in the patched‐1 (*PTCH1*) gene. *PTCH1* mutations are also described in patients with holoprosencephaly.

**Methods:**

We have established a locus‐specific database for the *PTCH1* gene using the Leiden Open Variation Database (LOVD). We included 117 new *PTCH1* variations, in addition to 331 previously published unique *PTCH1* mutations. These new mutations were found in 141 patients who had a positive *PTCH1* mutation analysis in either the VU University Medical Centre (VUMC) or Maastricht University Medical Centre (MUMC) between 1995 and 2015.

**Results:**

The database contains 331 previously published unique *PTCH1* mutations and 117 new *PTCH1* variations.

**Conclusion:**

We have established a locus‐specific database for the *PTCH1* gene using the Leiden Open Variation Database (LOVD). The database provides an open collection for both clinicians and researchers and is accessible online at http://www.lovd.nl/PTCH1.

## 1 INTRODUCTION

Basal cell nevus syndrome (BCNS, MIM#109400) or Gorlin syndrome is a rare autosomal dominant disorder characterized by multiple basal cell carcinomas (BCCs), maxillary keratocysts, and cerebral calcifications (John & Schwartz, 2016). The incidence of BCNS is estimated at 1 in 50,000 to 256,000 (Lo Muzio, 2008). Diagnostic criteria for BCNS were first established by Evans et al., (1993); modified by Kimonis et al., (1997) and revised in 2011 by Bree & Shah, (2011). Diagnosis is based on two major criteria, one major criterion and two minor criteria or one major criterion and genetic confirmation (Table 1).

**Table 1:** Diagnostic criteria for basal cell nevus syndrome (Bree & Shah, 2011). Two major criteria, one major criterion and two minor criteria, or one major and genetic confirmation is required for diagnosis

Major criteria	Minor criteria
Multiple BCCs or one BCC in a person younger than 20 years	Bifid, fused or splayed ribs
Odontogenic keratocysts	Other specific skeletal and radiologic abnormalities (i.e., pectus excavatum, scoliosis, hemivertebrae, Sprengel's deformity, syndactyly of digits, bony bridging of the sella turcica, flame‐shaped lucencies of phalanges)
Palmar or plantar pits	Macrocephaly
Lamellar calcification of the falx cerebri	Cleft lip or palate
Medulloblastoma in early childhood	Ovarian or cardiac fibroma
First‐degree relative with BCNS	Lymphomesenteric cysts
	Ocular anomalies (i.e., congenital cataract, coloboma, glaucoma, hypertelorism)

Most frequently occurring symptoms are multiple basal cell carcinomas, maxillary keratocysts, palmoplantar pits, and calcification of the falx cerebri. About 60% of patients have a typical phenotype with macrocephaly, frontal bossing, coarse facial features, and hypertelorism (Evans et al., 1993; Kimonis et al., 1997), 3%–5% of patients develop medulloblastoma in childhood (Evans, Farndon, Burnell, Gattamaneni, & Birch, 1991).

In 1996, the *patched‐1* (*PTCH1*) gene (MIM#601309) was first reported as a candidate gene for BCNS. Two different heterozygous mutations in the *PTCH1* gene were identified in two patients with Gorlin syndrome (Johnson et al., 1996). Another disorder that is caused by a germline mutation in the *PTCH1* gene is holoprosencephaly‐7 (MIM#610828), a structural anomaly of the brain in which there is failed or incomplete separation of the forebrain early in gestation. In addition, the vast majority of sporadic BCCs have somatic mutations in *PTCH1* (Bonilla et al., 2016; Reifenberger et al., 2005).

### 1.1 The *PTCH1* gene

*PTCH1* (NCBI Reference Sequence NM_000264.3) is the human homolog of the *Drosophila patched‐1* gene and is located on chromosome 9q22.3. It contains 24 exons with the transcriptional start site in exon 1 and the termination site in exon 23. *PTCH1* encodes a 1447‐amino acid transmembrane glycoprotein, which is part of the hedgehog (Hh) pathway. The Hh pathway is a key regulator in embryonic development and tumorigenesis controlling cell differentiation, tissue polarity, and cell proliferation.

The function of the PTCH1 protein is inhibition of the transmembrane protein Smoothened (SMO). Extracellular Hh ligands can bind to the PTCH1 receptor, releasing this inhibition, allowing SMO to signal downstream and activate GLI transcription factors. Based on this role in preventing cells from uncontrolled proliferation, *PTCH1* is seen as a tumor suppressor gene. *SMO* on the other hand acts as an oncogene (Kogerman et al., 2002).

The typical congenital features of BCNS seem to occur due to haploinsufficiency (Wicking et al., 1997), while tumors in BCNS are believed to develop according to the two‐hit hypothesis described by Knudson, (2001)and Pan, Dong, Sun, & Li, (2010). In the latter, either both alleles of the gene harbor a mutation, or one mutated allele is accompanied by allelic loss of the remaining wild‐type allele. Recent mouse model studies show that haploinsufficiency of *PTCH1* may be sufficient for the development of medulloblastoma and rhabdomyosarcoma, so tumor formation not always follows the two‐hit hypothesis (Calzada‐Wack et al., 2002; Zurawel, Allen, Wechsler‐Reya, Scott, & Raffel, 2000). With DNA sequencing analysis of the *PTCH1* gene, mutation detection frequency ranges from 50% to 85% in individuals with typical findings of BCNS (Lam, Ou, & Billingsley, 2013). Mosaic presentations of BCNS can occur (Reinders et al., 2016; Torrelo et al., 2013).

### 1.2 Ethical compliance

Our study was approved by the independent ethics committee of our hospital.

### 1.3 The *PTCH1* database

We have established a database for *PTCH1* using the Leiden Open Variation Database (LOVD) version 3.0 (Variants of patched 1 (PTCH1), 2004). The purpose of this database is to assemble molecular variants of the *PTCH1* gene in a standardized format. The database provides an open collection for both clinicians and researchers containing published and unpublished *PTCH1* mutations.

For each mutation, information is provided at the molecular level: DNA change, predicted protein change, RNA change, exon, type of mutation, reported pathogenicity, technique used, and source of material, and phenotype information if available. The Sequence Variant Nomenclature of all mutations (new and published) is updated according to the latest guidelines of the Human Genome Variation Society (HGVS) version 15.11 and based on NCBI Reference Sequence NM_000264.3.

## 2 ANALYSIS OF THE DATABASE

The database lists 331 previously published unique *PTCH1* mutations (http://www.lovd.nl/PTCH1). In addition, we included 117 new *PTCH1* variations (Table 2). These mutations were found in 141 patients who had a positive *PTCH1* mutation analysis in either the VU University Medical Centre (VUMC) or Maastricht University Medical Centre (MUMC) between 1995 and 2015. Mutation analysis was performed using Sanger sequencing and Multiplex Ligation‐dependent Probe Amplification. Polymorphisms and patients from the same family were excluded. Of the 117 different *PTCH1* variations, 110 mutations are classified as pathogenic or probably pathogenic according to the guidelines of the Associations for Clinical Genetic Science, the Dutch Society of Clinical Genetic laboratory Specialists, and the American College of Medical Genetics and Genomics (ACMG) (Wallis et al., 2013; Richards et al., 2015). A number of 23 *PTCH1* mutations are reported by previous studies and 79 are novel.

**Table 2:** New *PTCH1* mutations

Case identifier	Members affected	Gender	Age of diagnosis	Exon/Intron	DNA variant	Protein change	RNA change	Classification
Nonsense mutations								
BCNS1	1	M	67.7	2	c.205A>T	p.(Lys69*)	r.(?)	5
BCNS2	1	F	24.4	2	c.279C>G	p.(Tyr93*)	r.(?)	5
BCNS3	1	F	27.9	2	c.294C>A	p.(Cys98*)	r.(?)	5
BCNS4	2	M	38.9	3	c.403C>T	p.(Arg135*)	r.(?)	5
BCNS5	3	F	26.1	3	c.403C>T	p.(Arg135*)	r.(?)	5
BCNS6	3	M	11.0	3	c.466C>T	p.(Gln156*)	r.(?)	5
BCNS7	1	M	14.1	5	c.707G>A	p.(Trp236*)	r.(?)	5
BCNS8	2	U	56.6	8	c.1081C>T	p.(Gln361*)	r.(?)	5
BCNS9	1	F	36.7	8	c.1093C>T	p.(Gln365*)	r.(?)	5
BCNS10	1	M	8.4	8	c.1119C>G	p.(Tyr373*)	r.(?)	5
BCNS11	1	F	1.1	8	c.1198C>T	p.(Gln400*)	r.(?)	5
BCNS12	1	F	8.3	10	c.1379G>A	p.(Trp460*)	r.(?)	5
BCNS13	2	U	31.0	10	c.1380G>A	p.(Trp460*)	r.(?)	5
BCNS14	1	M	0.0	12	c.1691T>G	p.(Leu564*)	r.(?)	5
BCNS15	1	M	31.1	13	c.1804C>T	p.(Arg602*)	r.(?)	5
BCNS16	1, mosaicism	F	23.0	13	c.1810G>T	p.(Glu604*)	r.(?)	5
BCNS17	1	F	33.0	14	c.1975C>T	p.(Gln659*)	r.(?)	5
BCNS18	2	F	24.4	14	c.2098C>T	p.(Gln700*)	r.(?)	5
BCNS19	1	F	25.1	14	c.2170G>T	p.(Glu724*)	r.(?)	5
BCNS20	2	F	56.7	15	c.2308C>T	p.(Arg770*)	r.(?)	5
BCNS21	1	M	42.4	15	c.2359G>T	p.(Glu787*)	r.(?)	5
BCNS22	1	F	11.5	15	c.2446C>T	p.(Gln816*)	r.(?)	5
BCNS23	1	M	19.7	15	c.2557C>T	p.(Gln853*)	r.(?)	5
BCNS24	1	M	4.3	16	c.2619C>A	p.(Tyr873*)	r.(?)	5
BCNS25	1	M	25.8	16	c.2619C>A	p.(Tyr873*)	r.(?)	5
BCNS26	1	M	14.1	16	c.2619C>G	p.(Tyr873*)	r.(?)	5
BCNS27	1	F	6.2	18	c.3027C>A	p.(Tyr1009*)	r.(?)	5
BCNS28	2	F	47.9	18	c.3027C>G	p.(Tyr1009*)	r.(?)	5
BCNS29	1, de novo	F	14.1	18	c.3058C>T	p.(Gln1020*)	r.(?)	5
Missense mutations								
BCNS30	1, de novo	F	28.8	4	c.591G>C	p.(Trp197Cys)	r.(?)	5
BCNS31	1	M	11.7	5	c.689C>G	p.(Thr230Arg)	r.(?)	4
BCNS32	1	M	31.1	6	c.890T>C	p.(Leu297Pro)	r.(?)	4
BCNS33	2	M	7.4	10	c.1439C>T	p.(Ser480Leu)	r.(?)	4
BCNS34	1	M		10	c.1450G>A	p.(Gly484Arg)	r.(?)	4
BCNS35	1	M	36.9	11	c.1526G>A	p.(Gly509Asp)	r.(?)	5
BCNS36	1	M	58.4	11	c.1526G>A	p.(Gly509Asp)	r.(?)	5
BCNS37	3	M	15.1	11	c.1526G>A	p.(Gly509Asp)	r.(?)	5
BCNS38	1	F	40.3	11	c.1555G>C	p.(Ala519Pro)	r.(?)	4
BCNS39	1	U	39.2	12	c.1712G>C	p.(Arg571Pro)	r.(?)	4
BCNS40	1	M	38.9	14	c.2250G>C	p.(Lys750Asn)	r.spl?	4
BCNS41	1	M	46.7	15	c.2414T>G	p.(Ile805Arg)	r.(?)	4
BCNS42	1	M	22.6	15	c.2447A>G	p.(Gln816Arg)	r.(?)	4
BCNS43	1	F	52.1	18	c.2917C>A	p.(Gln973Lys)	r.(?)	4
Splice site mutations								
BCNS44	1	F	17.1	1i	c.202‐2A>G	p.?	r.spl?	4
BCNS45	2	F	23.5	2i	c.394+1G>A	p.?	r.spl?	4
BCNS46	2	M	17.7	2i	c.394+1G>C	p.?	r.spl?	4
BCNS47	1	F	13.6	4i	c.566_584+8del	p.?	r.spl?	4
BCNS48	1	M	12.3	4i	c.655‐1G>A	p.?	r.spl?	4
BCNS49	1	F	21.5	5i	c.747‐2A>G	p.?	r.spl?	4
BCNS50	4	M	2.6	6i	c.946‐1G>T	p.?	r.spl?	4
BCNS51	2	F	22.3	8i	c.1216‐2A>G	p.?	r.spl?	4
BCNS52	2	F	33.1	9i	c.1347+1G>A	p.?	r.spl?	4
BCNS53	2	M	32.3	9i	c.1348‐1G>C	p.?	r.spl?	4
BCNS54	1	M	24.0	10i	c.1504‐1G>C	p.?	r.spl?	4
BCNS55	1	M	59.7	10i	c.1504‐2A>T	p.?	r.spl?	4
BCNS56	2	M	27.1	12i	c.1729‐1G>C	p.?	r.spl?	5
BCNS57	1	M	69.4	14i	c.2250+1G>T	p.?	r.spl?	4
BCNS58	1	F	28.2	14i	c.2251‐2A>G	p.?	r.spl?	4
BCNS59	1	M	7.1	15i	c.2561‐2A>G	p.?	r.spl?	4
Small deletions or duplications								
BCNS60	1, de novo	M	5.4	1	c.114del	p.(Leu39Cysfs*41)	r.(?)	5
BCNS61	2	M	31.0	2	c.254_255del	p.(Arg85Thrfs*4)	r.(?)	5
BCNS62	1	F	49.7	2	c.258_259del	p.(Leu87Ilefs*2)	r.(?)	5
BCNS63	1	M	28.5	2	c.258_259del	p.(Leu87Ilefs*2)	r.(?)	5
BCNS64	1	M	52.0	2	c.262_266del	p.(Phe88Thrfs*50)	r.(?)	5
BCNS65	1	M	30.9	2	c.385_386dup	p.(Trp129Cysfs*9)	r.(?)	5
BCNS66	1	M	14.7	3	c.479_482del	p.(Gln160Profs*10)	r.(?)	5
BCNS67	1	M	10.7	3	c.572_575dup	p.(Met192Ilefs*61)	r.(?)	5
BCNS68	1	F	63.8	5	c.724del	p.(Gln242Serfs*8)	r.(?)	5
BCNS69	1	F	25.8	6	c.770_771delinsGGTTTGG	p.(Thr257Argfs*14)	r.(?)	5
BCNS70	1	F	43.8	6	c.842del	p.(Met281fs*2)	r.(?)	5
BCNS71	1	F	11.4	7	c.1040_1049del	p.(Val347Alafs*17)	r.(?)	5
BCNS72	1	F	35.1	8, 14	c.[1114del;2183C>T]	p.[(Met372Cysfs*60);p.(Thr728Met)]	r.(?)	5;3
BCNS73	1	F	23.9	9	c.1279del	p.(Leu427Trpfs*5)	r.(?)	5
BCNS74	1	M	35.1	10	c.1348_1350del	p.(Leu450del)	r.(?)	4
BCNS75	1	M	55.5	10	c.1366dup	p.(Thr456Asnfs*41)	r.(?)	5
BCNS76	2	F	13.3	10	c.1415_1429del	p.(Ala472_Leu476del)	r.(?)	4
BCNS77	1	M	47.9	11	c.1508dup	p.(Leu503fs*)	r.(?)	5
BCNS78	1	M	2.7	13	c.1767_1769del	p.(Leu590del)	r.(?)	4
BCNS79	1	F	8.3	14	c.1852del	p.(Cys618Alafs*5)	r.(?)	5
BCNS80	1	M	43.0	14	c.1925dup	p.(Pro643Thrfs*11)	r.(?)	5
BCNS81	1	F	16.4	14	c.2011dup	p.(His671Profs*10)	r.(?)	5
BCNS82	1	M	15.5	14	c.2178_2179insA	p.(Cys727Metfs*11)	r.(?)	5
BCNS83	1	M	14.9	14	c.2179del	p.(Cys727Valfs*19)	r.(?)	5
BCNS84	1	M	42.2	16	c.2612_2615del	p.(Asn871Ilefs*31)	r.(?)	5
BCNS85	1	F	9.7	17	c.2748del	p.(Ser917Alafs*7)	r.(?)	5
BCNS86	1	F	1.2	17	c.2793del	p.(Val932Serfs*30)	r.(?)	5
BCNS87	1	F	14.5	17	c.2833_2843del	p.(Arg945Glyfs*10)	r.(?)	5
BCNS88	1	M	52.4	18	c.3050del	p.(Phe1017Serfs*32)	r.(?)	5
BCNS89	2	M	40.3	18	c.3056_3059del	p.(Glu1019Glyfs*29)	r.(?)	5
BCNS90	2	F	21.0	18	c.3107_3108del	p.(Leu1036Cysfs*108)	r.(?)	5
BCNS91	3	F	0.6	18, 23	c.[3135del;4048C>T]	p.[(Phe1046Serfs*3;(Arg1350Trp)]	r.(?)	5;3
BCNS92	1	M	15.8	18	c.3139_3142del	p.(Leu1047*)	r.(?)	5
BCNS93	1	M	13.8	18	c.3139del	p.(Leu1047fs*11)	r.(?)	5
BCNS94	1	F	23.1	18	c.3150del	p.(Trp1051fs*7)	r.(?)	5
BCNS95	1	M	33.2	19	c.3233_3239del	p.(Leu1078Profs*7)	r.(?)	5
BCNS96	1	U	9.3	19	c.3251_3272del	p.(Val1084Alafs*2)	r.(?)	5
BCNS97	1	M	44.7	20	c.3364_3365del	p.(Met1122Valfs*22)	r.(?)	5
BCNS98	1	M	10.9	20	c.3364_3365del	p.(Met1122Valfs*22)	r.(?)	5
BCNS99	1	F	54.4	20	c.3375del	p.(Val1126Serfs*13)	r.(?)	5
BCNS100	1	F	41.8	21	c.3497dup	p.(Asn1166Lysfs*18)	r.(?)	5
BCNS101	1	U	6.7	21	c.3525_3526del	p.(Leu1175Phefs*8)	r.(?)	5
Large deletions or duplications								
BCNS102	1	F	48.5	_1_24_	c.(?_‐188)_(*3411_?)del	p.0?	r.0?	5
BCNS103	1	M	47.2	_1_24_	c.(?_‐188)_(*3411_?)del	p.0?	r.0?	5
BCNS104	1	F	14.2	_1_24_	c.(?_‐188)_(*3411_?)del	p.0?	r.0?	5
BCNS105	1	M	36.6	_1_2i	c.(?‐188)_(394+1_395‐1)del	p.?	r.(?)	5
BCNS106	1	F	38.0	2i_12i	c.(394+1_395‐1)_(1728+1_1729‐1)(2)	p.?	r.(?)	4
BCNS107	1, de novo	F	14.9	2i_16i	c.(394+1_395‐1) _(2703+1_2704‐1)(2)	p.?	r.(?)	4
BCNS108	1	F	37.1	2i_24_	c.(394+1_395‐1)_(*3411_?)del	p.?	r.(?)	5
BCNS109	1	F	19.9	14i_16i	c.(2250+1_2251‐1)_(2703+1_2704‐1)del	p.?	r.(?)	5
BCNS110	4	M	35.0	16i_19i	c.(2703+1_2704‐1)_(3306+1_3307‐1)del	p.?	r.(?)	5
Probably nonpathogenic								
BCNS111	1	F	27.3	7i	c.1067+5G>C	p.?	r.spl?	2
BCNS112	1	F	51.2	13	c.1792A>T	p.(Met598Leu)	r.(?)	2
BCNS113	1	M	70.3	14	c.2173C>T	p.(Pro725Ser)	r.(?)	2
BCNS114	1	F	33.7	18	c.3155C>T	p.(Thr1052Met)	r.(?)	2
BCNS115	1	M	14.0	18	c.3155C>T	p.(Thr1052Met)	r.(?)	2
BCNS116	2	F	43.3	18	c.3155C>T	p.(Thr1052Met)	r.(?)	2
BCNS117	2	M	29.9	21	c.3487G>A	p.(Gly1163Ser)	r.(?)	2

NCBI Reference transcript PTCH1: NM_000264.3. All variants were detected in a heterozygous state (if two variants were present, they are presumed to be on the same allele, in trans). Variant nomenclature is according to HGVS guidelines. Classification is according to ACMG, ACGS, and VKGL criteria; class 5: pathogenic, class 4: likely pathogenic, class 3: uncertain significance, class 2: likely benign, class 1: benign.

Of the 110 new mutations 38% (42/110) are frameshift (33 small deletions, eight small duplications, one small indel), 26% (29/110) nonsense, 13% (14/110) missense, and 15% (16/110) are splicing mutations. The remaining 8% (9/110) are large genomic *PTCH1* deletions and duplications. The mutations were found in all coding exons (1–23) and no hotspot was found.

The majority of patients were from the Netherlands (68%, 76/110). The age of DNA test ranged from 0 to 70 years, with a mean age of 26.5 years. Motivations for genetic testing written on the application forms were: (1) clinical suspicion of BCNS (47.4%); (2) clinical diagnosis of BCNS (32.6%); (3) first‐degree family member with BCNS (16.8%); and (4) family members with symptoms of BCNS (3.2%). In total, four individuals were prenatally screened for BCNS, because of a first‐degree family member or a clinical suspicion based on ultrasonography.

## DATABASE AVAILABILITY

The data are accessible to the public at http://www.lovd.nl/PTCH1. Contributors will have to register for a login and password.

## CONFLICT OF INTEREST

The authors have no conflicts of interest to declare.
